# Hæmorrhagic Malarial Fever

**Published:** 1889-09

**Authors:** I. T. Young

**Affiliations:** La.


### Southern Medical Record.

A MONTHLY JOURNAL OF PRACTICAL MEDICINE.

☞ All communications must be addressed to R. C. WORD, Managing Editor.

"*Quicquid Præcipies Esto Brevis.*"

VOL. XIX ATLANTA, GA., SEPTEMBER, 1889. NO. 9

### ORIGINAL AND SELECTED ARTICLES.

### HÆMORRHAGIC MALARIAL FEVER.

BY I. T. YOUNG, M. D., OF LA.

I have been called upon by some of my professional brethren for my treatment of hæmorrhagic fever.

I have turned a deaf ear to their calls, some of them for several years; feeling that perhaps the future would bear me out in what the past had taught me to think on this subject; but after an experience of more than eleven years in the treatment of this disease, I feel that it is my duty to speak out if members of the profession wish to hear what I have to say.

As I have not anticipated writing, and hence have not kept notes, it will be impossible to write an article as precise or as interesting as it should be on such a subject; but to withhold what I may know when the information is sought, would be to shirk my duty.

With this apology I have resolved to say something on this important subject; for there seems to be, as yet, no accepted plan of treatment for this terrible scourge of the low land sections of our malarial country. Of the numerous articles I have read, no two of them agree.

The pathology of this disease must be before the mind of the practitioner before he can treat it rationally.

There has been enough careful, scientific investigation on this subject, to direct the minds of the profession in one channel; but not so with the treatment; in this every man seems to differ from his neighbor.

Distressing nausea and vomiting, not retaining even a teaspoonful of water; voiding large quantities of bloody urine every few hours and sometimes every few minutes; feeble, wiry pulse, cold skin, pinched features, skin very yellow, patient very restless and chill coming on, in many instances, at irregular intervals; often pulse not perceptible at the wrist, and skin very cold.

This sums up some of the most formidable symptoms of this morbid condition known as hæmorrhagic fever or malarial hæmaturia. The usual tendency of the chill is a diurnal return; the fever is not always very high, but if the collapse does not persist, is frequently high, and there is much depression of the vital powers, which is frightful when the chill returns with its accompanying hemorrhage. No two cases have followed exactly the same course, yet, in general much the same; fever declining usually toward morning, and a return of chill followed by hemorrhage from kidneys, sometimes in the forenoon.

For the nausea and vomiting, for the chill, and to sustain the vital powers by stimulating the heart's action, I give from 5 to 10 drops of chloroform in a teaspoonful of glycerin and water, to be repeated at intervals as indicated; if a chill, or congestion is threatened I repeat every 15 or 20 minutes until circulation is re-established, or is partially restored, (for I have seen the circulation very feeble for days); I sometimes give much larger doses in the emergency, in which case I lengthen the intervals, taking care not to push it beyond its stimulating effect on the heart. I frequently use creosote and other anti-emetics to allay nausea, but in some of my worst cases chloroform has answered better while meeting more indications, which is quite an item with such a condition of the stomach; also to relieve this symptom, and to stimulate the liver I often prescribe calomel, but in broken doses, taking care not to purge.

For the hemorrhage I rely on ergot, in which I have great faith; some ask why not other hemostatic as well—perhaps they may do as well, but I have tried the ergot and I have not tried them; I believe however, that ergot is peculiarly appropriate for this trouble on account of its action on the vaso motor system, its peculiar action on the arterioles, making it especially indicated in this malady; not only does it produce contraction of the arterioles, but I think it tones up the vascular system when persisted in, as well as preventing and arresting hemorrhage, (thus counteracting to a great extent some of the evils of quinine.)

I think it is a great mistake in this malady, to lose sight of the other qualities of ergot, and use it only as a hemostatic, thus leaving it off as soon as the hemorrhage is arrested, which often proves only temporary; the hemorrhage returning with the next chill worse than the first. The ergot should be given in teaspoonful doses (fluid ext.) repeated as often as indicated, to arrest the hemorrhage, and then continued in smaller doses or at long intervals for several days, or even longer, after the hemorrhage has been arrested.

It seems strange that patients who cannot retain a spoonful of water will retain this dose.

I have found it necessary, however, to be very energetic and persistent to accomplish this in some cases, first to settle their stomach as much as possible, by appropriate treatment, before giving the ergot; and next by using cold water sprinkled in their faces, wet towels, and sometimes ice, sinapisms, &c. Believe it *can* be done before you begin, and you will accomplish it.

To keep off an expected chill is a matter of great importance; of so much importance that the physician is apt to lose sight of other important facts in the case; the fact that the patient's condition contraindicates quinine is the first.

Here with a patient who has but few healthy red blood corpuscles left in the circulation, whose vital powers are so low that their re-formation is much interfered with, and with hemorrhages from the kidneys, the organ which eliminates the great bulk of the quinine taken into the system.

In this condition what of quinine? Here is quinine with a theory that it retards the re-formation of red blood corpuscles, that it interferes with the functions of the few left in the circulation, that it dilates the arterioles and promotes hemorrhage, that it is not readily eliminated when the kidneys are affected &c., and with a fact (so accepted) that it prevents the return of the chill, and if the chill is prevented the patient's chances are much improved, if not made absolutely good. There are two sides to every question; the theory is that it will do harm; the fact is that it is very uncertain in its antiperiodical effect in this malady, usually fails to keep off the chill while aggravating the symptoms of the patient; aggravating the nausea, the restlessness and the hemorrhage; and in some cases it absolutely produces hemorrhage even in a single large dose.

"A very rapid disintegration of red corpuscles appears sometimes to take place when a morbid poison is present in the blood." (Principles Human Phys., Carpenter, page 203, paragraph 190). Might not quinine, given in large doses, produce this effect when the kidneys fail to perform their functions? We know that it is not changed in the system, and must be eliminated, chiefly through the kidneys and hence must accumulate in the blood when the kidneys fail to perform their functions.

In many cases as there is no assimilation it acts only as an irritant to the stomach.

Some will say, well, what would you do then? you would give no quinine.

I give it, but very cautiously, and never in large doses, as I so often do in other remittent fevers of malarial origin; and the reverse to the ergot, I discontinue as soon as I can (until after convalescence begins, when I use a small quantity in an iron tonic).

In the first place there is very little assimilation and it is very uncertain about getting it appropriated in time to meet the indications.

I find that piperine to ward off the chill and thus prevent congestion, and hence prevent the hemorrhage, is much more certain than quinine, and then it promotes the assimilation of quinine when combined with it.

I usually give 2 grains of quinine combined with 2 grains of piperine every two hours until 4 or 5 doses are taken before the chill time, and always give a dose of ergot before an expected chill.

In some cases I have used Fowler's solution with apparently good results (in cases when quinine and piperine could not be retained by the stomach) at least my patient recovered. I have never tried, however, to treat a case from the beginning to the completion of convalescence without quinine (except perhaps in one case in which I substituted arsenic. I have treated them until they were out of bed without quinine, but used it during convalescence) nor do I think I would like to do so, recognizing, as I do, that the malarial poison in the system is the foundation of the pathological condition with which we have to deal, and that quinine approaches nearer to a specific than any known remedy for this poison.

But I do believe that the delusion which controls the minds of many of the profession; that the foundation must be removed first, and then the superstructure, has sent many a poor sufferer to his long home.

We cannot always remove the cause before we attend to the resulting injuries. As well might we, when called to a soldier who is wounded by the shivered timbers from his stockade, and whose life blood is flowing from large severed arteries, say we must remove the cause, and begin to cut out the fragments of the timbers, while his life is ebbing away.

Would it not be better to ligate the arteries and to place our patient in the best condition we could to withstand the shock; and then proceed to remove the cause? As to remove the fragments of timber would tend to aggravate the hemorrhage while removing the cause, so does quinine tend to aggravate the hemorrhage (and other symptoms) while removing the malarial poison which is the cause of the condition upon which the hemorrhage depends.

It is not a question how much quinine the patient needs, but how much can he bear, which we are to consider. Assimilation is so bad in these cases that I would not trust capsules, but would make fresh pills with glycerin and water, (when it cannot be taken in solution) these will often fail to keep off the chill; and then when the first symptom of the chill is felt, begin with chloroform as before directed, first having given ergot (fluid ext.) a teaspoonful in water.

While in many cases the depressed condition (I might say collapse) occurring with the chill and hemorrhage is very persistent and lasts for days, needing constant and close attention, in others after a time the fever rises and we need appropriate treatment to keep down the temperature; but should guard against cardiac depressors, or combine them with cardiac stimulants; I don't confine myself to any one of this class of remedies, but proceed with caution, always trying the milder first. I frequently prescribe sweet spts. nitre combined with digitalis, as safe, and often meeting more than one indication.

Mild diuretics, as far as I can judge, have acted beneficially in this trouble, and are not detrimental as some seem to think.

Another important point is yet to be considered; that of sustaining the vital powers, which we always find low, and usually *very* low.

This is a task by no means easy, and yet, it *must* be done.

Some cases, I find, cannot retain a bit of nourishment, except a very small quantity of Ducro's Alimentary Elixir every two hours, diluted with a little water. I have seen patients who could take no other nourishment for days, and having frequent and copious hemorrhages a part of that time.

I give them as soon as I can get them to retain it, a tablespoonful of a solution containing ʒ i. of carbonate of ammonia to ℥ x of water, (or 3 grains to a dose) followed by two tablespoonfuls of sweet milk, every 2 or 3 hours as the condition of the patient will permit, (in some cases less milk as the stomach will reject that quantity.)

And sometimes, especially if much prostration, will alternate with Ducro's elixir. I give as generous a diet as I can when my patient will take it, but the ammonia and milk is forced, unless persistently rejected by the stomach : in such cases the Ducro is given and usually retained. Nourishment at intervals of two hours is best in these cases where the quantity borne by the stomach is so small.

I always give carbonate of ammonium when my patient can take it. I think it is clearly indicated for its effects on the circulation, on the nervous system, and for the gastric disturbance; add to this its effects as an alkali (for some advocate alkalies very strongly in this affection) and it meets many indications ; and my experience has led me to set some value on it as an antiperiodic, and to hope that it will in some measure atone for the deficiency in the dose of quinine used. In fact I have left off quinine entirely in some cases, its effects have been so bad, and have used no antiperiodic, unless the ammonia should be so regarded, and my patients have done much better without it than with it (using a little quinine during convalescence in these cases). I usually give tinct. of iron as soon as the stomach is in a condition to bear it; or give a tonic containing iron in some form. It is often impossible to follow a prescribed course owing to the intolerance of the stomach; and we must use our discretion as to what we can best leave off.

With this general line of treatment and a careful attention to symptoms, and good nurses to back me, I have had the privilege of seeing every one of my patients (which have not been a few) get on their feet again; none, with this form of malaria having died on my hands during a practice of eleven years.

My friend and relative, Dr. T. W. Lilley from whom I obtained many of my ideas of the treatment of this disease, had the same success, saying (when I conversed with him on this subject, a short time before his death, which occurred in 1879 from yellow fever) he was curing all his cases.

I do not presume to think that I shall always be so successful, but a treatment which has stood the test for eleven years, besides the years a similar treatment was tested in the hands of another, ought to be worthy of a trial by the profession, especially in a disease so dreaded as the one in question.

I have not, however, learned to feel that a patient is safe in my hands with this disease, and will feel an inclination to shrink from the responsibility when called to a case, especially those cases who have had it before. I feel that I cannot close without reference to the subsequent history of these cases.

Assuming at once, on rational grounds, that it would not do for a patient who had been afflicted with this disease to remain another summer exposed to the same influences to which the resisting powers of his system had proved so inadequate, I advised all my patients who had contracted the disease at their own homes to change their domicil before the next summer, in fact to go elsewhere to complete convalescence, and not to return except in winter. I have lost sight of a number of the cases, and especially those of a milder type; but a number of the most severe cases I have made it my business to keep up with, and in every case in which they refused to obey my instructions (for my advice was professed and urged) they have fallen a victim to this or some other malady within five years from their return to their old home, (the details of some of these cases would make a touching story) while they remained in a different part even of the same community they enjoyed reasonally good health, but a return to their old home would seldom permit them to pass more than one summer without a return of the disease, or some other severe form of malaria. I have been called to treat some of these cases the second, the third, and some of them even the fourth time at intervals of one and two years, and as they convalesced would repeat the admonition, not to stay in that place another summer, (those who have subsequently died were not under my care when they died.)

I am much inclined to the belief, that no patient who has had a severe attack of this disease is ever sound again; although he may be in apparently good health; that he will not withstand a severe attack of any malady as a healthy person would stand it, and I am certain that they are much more susceptible to malarial influences. And now, in conclusion, I will say to those who read this article; if you expect to have easy sailing while you cure your patients by this treatment, you will certainly be disappointed.

You must give your patient close and unremitting attention, you must have good nurses, and see to it that they obey your orders and carry out your designs; (it is certainly a mistake to expect untrained nurses to attend to such important duties without oversight, the correct performance of which would require judgment and intellect equal to, if not superior to the doctor) you must treat symptoms when necessary, always bearing in mind the pathological condition of your patient, and be careful not to depress, but to support and sustain the vital powers.

That which you cannot do for your patient, be sure you do not prevent nature from doing for him.

Do this and I will be glad to hear from the results.

